# Impact of the *sickle* mutant and temperature on the structure of transcripts and RNAs from *Arabidopsis thaliana*

**DOI:** 10.1186/s13104-022-05963-y

**Published:** 2022-03-22

**Authors:** Carine M. Marshall, Frank G. Harmon

**Affiliations:** 1 Plant Gene Expression Center, USDA-ARS, Albany, 94710 USA; 2 Department of Plant and Microbial Biology, University of California, Berkeley, CA 94720 USA; 3 Present Address: Department of Plant Biology, University of California, Davis, CA 95616 USA

**Keywords:** *Arabidopsis thaliana*, FASTQ file, Illumina sequencing, mRNA splice variant, RNAseq, SICKLE, Temperature response

## Abstract

**Objectives:**

The objective of this data set was to identify how interaction between temperature and the *sickle-3* (*sic-3*) mutant alters the global messenger RNA (mRNA) content of *Arabidopsis thaliana* seedlings. The motivation was discovery of atypical mRNA splice variants in *sic-3* that differed with seedling growth temperature. The expected outcome was identification of mRNA splice variants altered by *sic-3*, temperature, or the combination of temperature and genotype.

**Data description:**

The data set is RNAseq profiling of Arabidopsis (Col-0 ecotype) wild type and *sic-3* seedlings under 16 °C or 28 °C. A comprehensive view of global mRNA sequences and their content was captured by deep sequencing of RNA pools made from sets of seedlings sampled every 4 h over 20 h. This data set contains sequences representing the spectrum of mRNA splice variants from individual genes, as well as from mRNA-related sequences like spliced introns. This data set enables detection of significant changes in gene-level expression and relative levels of mRNA splice variants caused by the different growth temperatures, the *sic-3* mutation or both factors. This data set is useful to study production of mRNA splice variants and other mRNA-related RNAs in a range of plant species because Arabidopsis is a model plant.

## Objective

The objective of this RNAseq data set was to identify how the growth temperatures 16 °C and 28 °C alone or in combination with the *sickle-3* (*sic-3*) mutant alter the global messenger RNA (mRNA) content of *Arabidopsis thaliana* seedlings. This work was motivated by a prior study demonstrating certain mRNA splice variants accumulate in the *sic-3*, an effect modified by exposure of seedlings to either 16 °C or 28 °C [1]. To capture a comprehensive view of global mRNA sequence content, RNA isolated from seedlings collected every 4 h over a 20-h period was pooled together and this pool deep sequenced by Illumina HiSeq4000 paired-end sequencing. The experimental design focused on identification and quantification of the spectrum of mRNA splice variants arising from individual genes. This data set is also expected to contain other types of RNA sequences related to mRNAs, particularly spliced introns because plants carrying a second *sic* mutant allele have high levels of free intron sequences [2]. This data set enables discovery of significant differences in whole gene-level expression and in the relative levels of different mRNA splice variants between each combination of genotype and temperature condition. These data are useful for studies investigating the factors that shape the makeup of the mRNA pool in Arabidopsis and other plant species.

## Data description

The data set is RNAseq of whole Arabidopsis (Col-0 ecotype) seedlings of wild type at 16 °C (Table 1, data set 1–3) or 28 °C (Table 1, data set 4–6), or the *sic-3* mutant at 16 °C (Table 1, data set 7–9) or 28 °C (Table 1, data set 10–12). Samples were from 10-day-old seedlings grown on 1X Murashige and Skoog basal salt medium with micropropagation agar. Prior to collection, seedlings were grown under 12 h light (50 μmol photons m^−1^ s^−1^ from cool white fluorescent bulbs) and 12 h darkness at 22 °C. When seedlings reached 10 days old, they were transferred to constant light and either 16 °C or 28 °C at 12 h after the lights came on at dawn. Beginning at 20 h after dawn (i.e., 8 h after transfer to the new temperature), samples were collected every 4 h for the next 20 h. A total of 6 samples were collected over this time course. Three biological replicates were collected in this manner. Total RNA from these samples were pooled for each genotype and temperature combination. Strand-specific RNAseq libraries were prepared with the ScriptSeq v2 RNAseq library preparation kit from Epicentre and the libraries were indexed using ScriptSeq Index PCR primers from Epicentre. Libraries were paired-end sequenced at 150 base pairs by Illumina HiSeq4000. Sequencing reads were filtered with the Illumina quality filter, which requires that no more than 1 base call in the first 25 cycles has an Illumina chastity value less than 0.6. Pass-filter reads were those meeting the Illumina quality filter and these were demultiplexed according to the 12 biological samples into forward read and reverse read FASTQ files. The total number of pass-filter reads for each data set were as follows: 24,331,212 in data set 1, 27,630,232 in data set 2, 34,578,935 in data set 3, 29,286,330 in data set 4, 31,867,914 in data set 5, 29,843,882 in data set 6, 38,174,014 in data set 7, 28,862,139 in data set 8, 42,055,703 in data set 9, 32,372,213 in data set 10, 31,700,336 in data set 11, and 35,333,599 in data set 12. The FASTQ files were deposited as a BioProject at the National Center for Biotechnology (NCBI) under accession number PRJNA758710 (https://identifiers.org/ncbi/bioproject:PRJNA758710) [3].

**Table 1:** Overview of data files/data sets

Label	Name of data file/data set	File types (file extension)	Data repository and identifier (DOI or accession number)
Data set 1	RNAseq of Col-0 exposed to 16 °C, replicate 1	.FASTQ	NCBI Sequence Read Archive (https://identifiers.org/ncbi/insdc.sra:SRR15673685) [4]
Data set 2	RNAseq of Col-0 exposed to 16 °C, replicate 2	.FASTQ	NCBI Sequence Read Archive (https://identifiers.org/ncbi/insdc.sra:SRR15673684) [5]
Data set 3	RNAseq of Col-0 exposed to 16 °C, replicate 3	.FASTQ	NCBI Sequence Read Archive (https://identifiers.org/ncbi/insdc.sra:SRR15673681) [6]
Data set 4	RNAseq of Col-0 exposed to 28 °C, replicate 1	.FASTQ	NCBI Sequence Read Archive (https://identifiers.org/ncbi/insdc.sra:SRR15673677) [7]
Data set 5	RNAseq of Col-0 exposed to 28 °C, replicate 2	.FASTQ	NCBI Sequence Read Archive (https://identifiers.org/ncbi/insdc.sra:SRR15673676) [8]
Data set 6	RNAseq of Col-0 exposed to 28 °C, replicate 3	.FASTQ	NCBI Sequence Read Archive (https://identifiers.org/ncbi/insdc.sra:SRR15673675) [9]
Data set 7	RNAseq of *sic-3* exposed to 16 °C, replicate 1	.FASTQ	NCBI Sequence Read Archive (https://identifiers.org/ncbi/insdc.sra:SRR15673680) [10]
Data set 8	RNAseq of *sic-3* exposed to 16 °C, replicate 2	.FASTQ	NCBI Sequence Read Archive (https://identifiers.org/ncbi/insdc.sra:SRR15673679) [11]
Data set 9	RNAseq of *sic-3* exposed to 16 °C, replicate 3	.FASTQ	NCBI Sequence Read Archive (https://identifiers.org/ncbi/insdc.sra:SRR15673678) [12]
Data set 10	RNAseq of *sic-3* exposed to 28 °C, replicate 1	.FASTQ	NCBI Sequence Read Archive (https://identifiers.org/ncbi/insdc.sra:SRR15673674) [13]
Data set 11	RNAseq of *sic-3* exposed to 28 °C, replicate 2	.FASTQ	NCBI Sequence Read Archive (https://identifiers.org/ncbi/insdc.sra:SRR15673683) [14]
Data set 12	RNAseq of *sic-3* exposed to 28 °C, replicate 3	.FASTQ	NCBI Sequence Read Archive (https://identifiers.org/ncbi/insdc.sra:SRR15673682) [15]

## Limitations

- The *Arabidopsis* plants sampled were whole 10-day-old seedlings. This should be taken into account if these data are interpreted for plants at other stages of development or specific tissues.
- The pooling strategy taken here captures most mRNAs but mRNAs with low expression or expressed for a brief time may not be represented.
- While the read depth exceeds 20 million for all samples, low abundance mRNAs may not be well represented in the samples with lower read depth.

## Data Availability

All the FASTQ files described in this Data Note can be freely and openly accessed at the NCBI Sequence Read Archive (SRA) with the resolving links in Table 1. All data and details on the samples are gathered together under NCBI BioProject accession PRJNA758710 [3].

## Abbreviations

mRNA: Messenger RNA
NCBI: National Center for Biotechnology Information
SRA: Sequence Read Archive
*sic-3*: *sickle-3*
